# Understanding yellow fever-associated myocardial injury: an autopsy study

**DOI:** 10.1016/j.ebiom.2023.104810

**Published:** 2023-09-25

**Authors:** Fernando Rabioglio Giugni, Vera Demarchi Aiello, Caroline Silverio Faria, Shahab Zaki Pour, Marielton dos Passos Cunha, Melina Valdo Giugni, Henrique Trombini Pinesi, Felipe Lourenço Ledesma, Carolina Esteves Morais, Yeh-Li Ho, Jaques Sztajnbok, Sandra de Morais Fernezlian, Luiz Fernando Ferraz da Silva, Thais Mauad, Venâncio Avancini Ferreira Alves, Paulo Hilário do Nascimento Saldiva, Leila Antonangelo, Marisa Dolhnikoff, Amaro Nunes Duarte-Neto

**Affiliations:** a Departamento de Patologia, Faculdade de Medicina, Universidade de São Paulo, São Paulo, SP, Brazil; b Instituto do Coração InCor, Hospital das Clínicas HCFMUSP, Faculdade de Medicina, Universidade de São Paulo, São Paulo, SP, Brazil; c Laboratório de Evolução Molecular e Bioinformática, Instituto de Ciências Biomédicas, Universidade de São Paulo, SP, Brazil; d Departamento de Moléstias Infecciosas e Parasitárias, Faculdade de Medicina, Universidade de São Paulo, São Paulo, SP, Brazil; e Instituto de Infectologia Emílio Ribas, São Paulo, SP, Brazil; f Serviço de Verificação de Óbitos da Capital (SVOC), Universidade de São Paulo, São Paulo, SP, Brazil

**Keywords:** Yellow fever, 17DD, YEL-AVD, Myocarditis, Heart conduction system, Vascular endothelium, Cytokines, Chemokines, IP-10, Autopsy

## Abstract

**Background:**

Yellow fever (YF) is a viral hemorrhagic fever, endemic in parts of South America and Africa. There is scarce evidence about the pathogenesis of the myocardial injury. The objective of this study is to evaluate the cardiac pathology in fatal cases of YF.

**Methods:**

This retrospective autopsy study included cases from the São Paulo (Brazil) epidemic of 2017–2019. We reviewed medical records and performed cardiac tissue histopathological evaluation, electron microscopy, immunohistochemical assays, RT-qPCR for YF virus (YFV)-RNA, and proteomics analysis on inflammatory and endothelial biomarkers.

**Findings:**

Seventy-three confirmed YF cases with a median age of 48 (34–60) years were included. We observed myocardial fibrosis in 68 (93.2%) patients; cardiomyocyte hypertrophy in 68 (93.2%); endothelial alterations in 67 (91.8%); fiber necrosis in 50 (68.5%); viral myocarditis in 9 (12.3%); and secondary myocarditis in 5 (6.8%). Four out of five patients with 17DD vaccine-associated viscerotropic disease presented with myocarditis. The cardiac conduction system showed edema, hemorrhages and endothelial fibrinoid necrosis. Immunohistochemistry detected CD68-positive inflammatory interstitial cells and YFV antigens in endothelial and inflammatory cells. YFV-RNA was detected positive in 95.7% of the cardiac samples. The proteomics analysis demonstrated that YF patients had higher levels of multiple inflammatory and endothelial biomarkers in comparison to cardiovascular controls, and higher levels of interferon gamma-induced protein 10 (IP-10) in comparison to sepsis (p = 0.01) and cardiovascular controls (p < 0.001) in Dunn test.

**Interpretation:**

Myocardial injury is frequent in severe YF, due to multifactorial mechanisms, including direct YFV-mediated damage, endothelial cell injury, and inflammatory response, with a possible prominent role for IP-10.

**Funding:**

This study was funded by 10.13039/501100001807Fundação de Amparo à Pesquisa do Estado de São Paulo, 10.13039/100000865Bill and Melinda Gates Foundation, 10.13039/501100003593Conselho Nacional de Desenvolvimento Científico e Tecnológico, 10.13039/501100002322Coordenação de Aperfeiçoamento de Pessoal de Nível Superior.

**Research in context**

**Evidence before this study**

Yellow fever (YF) is a viral hemorrhagic fever, endemic in Africa and South America with high morbi-mortality. We searched MEDLINE and LILACS using the terms “yellow fever” AND “heart” OR “myocardium”, from database inception to April 2023, with no language restrictions. We excluded articles on non-human pathology and experimental studies. We found little evidence of heart involvement in YF. One cohort study showed a high rate of electrocardiographic abnormalities and bradycardia, with some findings suggesting myocarditis. Three pathology studies described alterations such as edema, hemorrhages and rare myocarditis, with detectable YF virus (YFV)-antigens in myocardium by immunohistochemistry. The remaining studies were case reports and small case series with brief descriptions of pathological findings.

**Added value of this study**

We performed an autopsy study that included 73 patients with confirmed YF. All cases died due to refractory shock with septic and hemorrhagic components, associated with liver failure, coagulopathy, hepatic encephalopathy, and acute kidney injury, during the São Paulo (Brazil) YF epidemics in 2017–2019. Most of them were men. We described the clinical and pathological features of the YF-associated myocardial injury and explored its mechanisms. We showed a high prevalence of endothelial abnormalities, interstitial edema and hemorrhages, cardiomyocyte hypertrophy and necrosis, myocardial fibrosis, and mononuclear myocarditis, attributed to YFV, in some cases, and to secondary myocardial infections (bacterial or fungal) in others. Four out of five patients with 17DD vaccine-associated viscerotropic disease presented with myocarditis. We found high levels of inflammatory and endothelial lesion biomarkers in situ. Elevated interferon gamma inducible protein-10 (IP-10) concentrations were remarkable in severe YF cases. We detected YFV components in the myocardium: YFV-RNA by RT-qPCR; YFV antigens by immunohistochemistry (within the cytoplasm of endothelial and inflammatory cells); and virus-like particles within endothelial cells cytoplasm through electron microscopy in 66, 24, and 1 examined cases, respectively.

**Implications of all the available evidence**

YF-associated myocardial injury is frequent in severe cases, has a myriad of pathological features and is associated with multifactorial mechanisms, including direct YFV damage, local and systemic inflammatory response, secondary bacterial and fungal sepsis, and endothelial and cardiomyocyte injury. The present findings may impact patient care by encouraging physicians to make a comprehensive cardiovascular evaluation in those patients, eventually leading to earlier therapeutic and supportive measures. Our results may also contribute to translational researchers investigating new biomarkers related to myocardial injury in YF, such as IP-10. YF is responsible for more than 20,000 deaths per year, mostly in low and mid-income countries, but has been devastating in high-income countries in the past. There is a risk of YF re-emergence in non-endemic, highly populated urban areas with many susceptible non-immunized people, as it happened in São Paulo, Southeastern Brazil, during the 2017–2019 epidemic. YF is a preventable disease with a highly efficient vaccine (17DD), but with a limited stockpile of it and an otherwise small therapeutic arsenal. On the other hand, the viral strain of the 17DD vaccine can cause end-organ dysfunction, not only to the liver and brain, but also to the heart, as demonstrated herein. A global coordinated effort is needed to support research and prevention of YF worldwide, and pathological studies, including those enrolling autopsies, are important to understand the mechanisms of injuries in different organs and systems.

## Introduction

Yellow Fever (YF) is a mosquito-borne viral hemorrhagic fever, considered a neglected tropical disease.^1^ It is endemic in parts of Africa and South America, with an estimated incidence of 200,000 cases per year and high lethality, which ranges from 20 to 50%.^2^^,^^3^ There are occasional outbreaks in non-endemic areas, such as the epidemics in southeastern Brazil from 2016 to 2019, which involved highly populated areas like the city of São Paulo, with a large number of non-immunized susceptible people.^4^ YF has an incubation time of 3–6 days, followed by a viremic phase when non-specific symptoms occur, including fever, myalgia, headache, nausea and malaise. This phase lasts a few days and most patients present remission of symptoms, but some individuals progress to a toxic phase, with severe multi-organ damage, such as shock, acute kidney injury, seizures, coma, gastrointestinal hemorrhages and especially acute hepatitis.^5^

In the mid-19th century, Jean Charles Faget described a relative bradycardia concurrent with fever in patients with YF, which became known as the Faget’s sign and was later seen in other infectious diseases. A prospective cohort study assessed the cardiovascular system of 70 patients hospitalized for YF and found a high rate of electrocardiographic (ECG) and echocardiographic abnormalities.^6^ One patient had a cardiac magnetic resonance suggestive of myocarditis.^6^ Other cohorts of hospitalized patients with YF described bradycardia, arrhythmias and myocarditis.^7^^,^^8^ Experimental studies have shown bradycardia, atrioventricular block and repolarization abnormalities in non-human primates infected with YF, with histopathological findings of cardiomyocyte degeneration, myolysis and, in few animals, lymphocytic perivascular inflammatory infiltrate in the myocardium.^9^^,^^10^ Regarding human pathology, non-specific findings were described in the heart of autopsied patients, such as edema, hemorrhages and hypertrophy.^11^^,^^12^ Myocarditis has been described in few studies.^6^^,^^12^, ^13^, ^14^ Other authors also reported positive immunohistochemistry (IHC) for viral antigens and positive reverse-transcriptase polymerase chain reaction (RT-PCR) for viral RNA in myocardial tissue.^13^^,^^15^, ^16^, ^17^, ^18^

Yet, the involvement of the heart in YF is poorly understood. Most autopsy studies are almost a century old and lack both systematic approaches and modern pathology techniques, while recent studies are mostly restricted to case reports and small case series. We performed an autopsy cohort study to assess the cardiac pathology of patients who died from YF in the 2017–2019 epidemics.

## Methods

### Design and inclusion

This is a retrospective autopsy cohort study. We included all patients with confirmed diagnoses of YF referred to the central morgue of the city of São Paulo—the Death Verification Service at the University of Sao Paulo–between 2017 and 2019. The majority of patients were hospitalized in Hospital das Clínicas da FMUSP, a tertiary academic hospital referenced for cases of YF, in which a crisis committee recommended performing autopsies in all deceased patients with YF. Autopsies were requested by assisting physicians from other hospitals when there was uncertainty about the cause of death. The definition of YF diagnosis required confirmation by serologic (positive IgM), molecular (RT-PCR) or tissue IHC methods in clinically suspected cases, according to the Brazilian authorities.^19^ Patients with YF vaccine-associated viscerotropic disease (YEL-AVD) were also included; the same diagnostic criteria were used, but with positive RT-PCR for vaccinal virus strain and negative for wild-type strain. We included patients who died from cardiovascular disease and sepsis from the Pathology Department archives as controls in IHC and proteomics analyses.

We collected demographic and clinical data from patients’ records. We retrieved their sex (assigned at birth), age, previous medical history, clinical and cardiovascular events during hospitalization, interventions, ECGs, echocardiograms and troponin.

### Autopsy and pathological assessment

All autopsies were performed by the Pathology Department of the University of Sao Paulo Medical School following the Letulle technique with systematic exam and representation of all organs. The hearts were externally examined, weighted, measured and sectioned. Tissue samples from left (LV) and right ventricle (RV) lateral walls were collected in all cases. In 8 patients, the cardiac conduction system was histologically represented. Collected tissues were fixed in formalin for 48 h and embedded in paraffin. Histological sections were performed and routinely stained with hematoxylin-eosin; other stains were performed at the pathologist’s discretion, such as Masson’s trichrome, Gram and Grocott stains. A tissue sample from the LV lateral wall was also immediately frozen in liquid nitrogen and stored in −80 °C freezers.

The histopathology of all samples was reviewed by a pathologist specialized in autopsy and infectious diseases pathology (ANDN) and a cardiologist (FRG); disagreements were resolved by consensus. A pathologist specialized in cardiovascular disease was consulted (VDA). Some histological features were systematically assessed in all cases: interstitial fibrosis, classified as perivascular, multifocal, and diffuse; cardiomyocyte hypertrophy, defined by enlarged, hyperchromic or misaligned nuclei; myocarditis, defined by inflammatory infiltrate associated with cardiomyocyte necrosis or degeneration; endothelial abnormalities, classified as mild in the presence of endothelial cells swelling, moderate if there was fibrinoid necrosis, and severe when vasculitis or thrombosis was found; interstitial hemorrhages, defined as the presence of red blood cells in the cardiac interstitium; fiber necrosis, defined as abnormalities compatible with cardiomyocyte death, such as loss of membrane integrity, dystrophic calcification, nuclear fragmentation, hypereosinophilia or contraction band necrosis; other findings considered to be relevant were reported as observed. We performed transmission electron microscopy (EM) in three selected cases.

### Immunohistochemistry

We performed IHC analysis in 24 YF cases: patients with histological viral myocarditis; cases with YEL-AVD; and youngsters without previous diseases and with clinical myocardial injury. We assessed YF viral antigen and classified it semiquantitatively as negative, positive 1+ and positive 2+. The primary antibody used in IHC reactions was an anti-YFV Mouse Ascitic Fluid (Immunoascite) specific to the virus (polyclonal anti-yellow fever virus) provided by Institute Pasteur-Dakar, originally standardized for enzyme-linked immunosorbent assay (ELISA) and validated in our laboratories at the optimized dilution of 1:20,000.^20^ For antibody validation, liver samples of YF confirmed cases were used as positive controls and negative YF cases, including biopsy and autopsy samples with different infectious diseases (viral and bacterial diseases) and steatohepatitis were used as negative controls. We also assessed CD68 (Agilent Cat# M0876, RRID:AB_2074844, 1:5000 dilution), CD45 (Agilent Cat# M070101-2, RRID:AB_2750582, 1:2000 dilution), CD57 (Agilent Cat# M7271, RRID:AB_2063218, 1:200 dilution), CD4 (Leica Biosystems Cat# NCL-L-CD4-368, RRID:AB_563559, 1:150 dilution), CD8 (Agilent Cat# M7103, RRID:AB_2075537, 1:400 dilution) by IHC in the same patients and in controls who died from cardiovascular disease and sepsis. Stained cells were counted in 30 high-power fields ( × 400) and comparisons were performed between YF patients and controls. We assessed the presence of angiopoietin-2 (Santa Cruz Biotechnology Cat# sc-74403, RRID:AB_1118956, 1:50 dilution) semiquantitatively by IHC, as 0 (absent), 1+ (<10%), 2+ (11%–50%) and 3+ (>50%), according to the number of stained vessels in the histological cut. In all IHC analyses, we used Mouse and Rabbit Specific HRP/DAB IHC Detection Kit–Micropolymer (ABCAM, AB_236466), cromogen diaminobenzidine (DAB), and Harris’ hematoxylin counterstaining.

### Viral RNA detection

We executed quantitative RT-PCR (RT-qPCR) for YF viral RNA in fresh frozen tissues collected from patients. Fragments measuring 1 cm³ were first macerated, then nucleic acid were extracted using guanidine thiocyanate (TRIzol, *Life Technologies*). Appropriate reagents for RT-qPCR were then used (AgPath-ID one-step RT–PCR reagents, *Ambion*) with primers and probes described in Supplementary Table S1. RT-qPCR reactions consisted of a reverse transcription step, at 45 °C for 10 min plus 95 °C for 10 min for enzyme activation, and 40 cycles at 95 °C for 15 s plus 60 °C for 45 s for hybridization and extension, using the ABI7500 equipment (*Thermo Fisher Scientific*).

The IHC, EM and RT-qPCR were performed as previously described.^18^

### Proteomics

We measured proteins related to the immune response (granulocyte-macrophage colony-stimulating factor [GM-CSF], interferon-gamma [IFN-γ], interleukin [IL]-10, IL-1β, IL-4, IL-6, IL-8, IL-12p70, interferon gamma-induced protein 10 [IP-10], monocyte chemoattractant protein-1 [MCP-1], macrophage Inflammatory Protein 1 Beta [MIP-1β], tumor necrosis factor-alpha [TNF-α]) and to the endothelial function (angiopoietin-2, endoglin, endothelin-1, vascular endothelial growth factor [VEGF], VEGF-A, VEGF-C, tissue factor, endocan, syndecan-1, thrombomodulin, troponin i, vascular cell adhesion protein 1 [VCAM-1], intercellular adhesion molecule 1 [ICAM-1], plasminogen activator inhibitor 1 [PAI-1], von Willebrand factor A 2 [vWF-A2] and fibrinogen) using multiplex immunoassay in fresh frozen tissue from patients with YF and from controls with cardiovascular disease and sepsis.

Fragments of approximately 10 mm³ of frozen tissue were processed according to instructions from the protein extraction kit (Bio-Plex Cell Lysis Kit–BIO-RAD, Cat# 171304011). Twenty-five microliters of tissue homogenate specimens were prepared for each analysis in 96-well plates utilizing the following kits: Milliplex Map Kit Human Cytokine/Chemokine Magnetic Bead Panel (Cat# HCYTMAG-60K), Milliplex Map Kit Human Angiogenesis/Growth Factor Magnetic Bead Panel 1 (Cat# MAGPMAG-24K), Milliplex Human Sepsis Magnetic Bead Panel 1 (Cat# HSP1MAG-63K), Milliplex Human Cardiovascular Disease Bead Panel 4 (Cat# HCY4MG-64K), Milliplex Map Kit Human Cardiovascular Disease Magnetic Bead Panel 3 Acute Phase (Cat# HCVD3MAG-67K) and Human Luminex Discovery Assay 7 PLEX—customized (Cat# LXSAHM-07), following the kit-specific protocols provided by Millipore (Millipore Corp., Billerica, MA, USA). Total protein was quantified by the automated colorimetric method using the proper kit (TP2, Cat# 4657586190, Roche Diagnostics GmbH, Mannheim, Germany) and equipment (COBAS C111, Roche Instrument Center, Rotkreuz, Switzerland). Analytes were quantified using a Magpix analytical test instrument, which utilizes xMAP technology, multiple analyte profiling, (Luminex Corp., Austin, TX), and xPONENT 4.2 software (Luminex). Concentrations of cytokines were determined on the basis of the fit of a standard curve for mean fluorescence intensity vs pg/ml and were divided by total protein for normalization.

### Statistics

We described qualitative data as frequency and proportions with 95% confidence intervals (using normal approximation), and quantitative data as mean and standard deviation (normally distributed data assessed by density plot and Shapiro–Wilk test) or median and interquartile range (non-normal data).

We compared the proportion of angiopoietin-2 positivity in IHC between groups using the Fisher exact test. We used the Wilcoxon rank-sum test for comparisons of cell counts in IHC and protein dosages between wild-type and YEL-AVD groups. We performed the Kruskal–Wallis test for comparisons between YF, sepsis and cardiovascular death in IHC cell counts and proteomics, followed by a post-hoc Dunn test for pairwise comparisons (with Benjamini-Hochberg adjustment) when statistical difference was detected. A p-value lower than 0.05 was considered statistically significant. We removed missing data from the analyses. Statistical analysis was performed in R version 4.1.2 and graphs were generated using the ggplot2 package.

### Ethics

This study was approved by the HC-FMUSP Ethical Committee and Institutional Review Board (protocol number 4.354.375). Patients’ next-of-kin gave written consent for the autopsy procedure.

### Role of funders

The funding sources had no involvement in this study.

## Results

A total of 696 cases of YF were reported in the State of São Paulo between 2017 and 2019, with 232 deaths (33.3%).^21^ Among those, 73 patients were referred to autopsy in São Paulo central morgue and included in this study. Supplementary Fig. S1 shows the inclusion flow diagram.

### Clinical characteristics

We included 68 (93.2%) patients with wild-type YF and 5 (6.8%) with YEL-AVD. 62 (84.9%) were male and the median age was 48 (34–60). The most prevalent comorbidity was hypertension (28.8%); only 5 (6.8%) patients had previous cardiac disease. Alcohol use (50.7%) and smoking (37.0%) were frequent. Patients had a median duration of hospitalization of 5 days and a median interval from symptoms to death of 9 days. All patients had shock requiring vasopressors, and 4 (5.5%) received inotropes. Supraventricular tachyarrhythmias (20.5%), bradyarrhythmias (6.8%) and Faget sign (11.0%) were reported. Forty-two (57.5%) patients had at least one ECG performed, considered normal in 8 (19.0%) of them. Non-sinus rhythm (42.9%), repolarization abnormalities (29.3%) and sinus bradycardia (26.9%) were the most frequent findings. Twenty-four (32.9%) patients had an echocardiogram performed, of which 3 (12.5%) presented with LV dysfunction; all had previous cardiac disease. Fifty-eight (79.5%) patients had troponin measurements; 54 (93.1%) had at least one concentration greater than the upper limit of normal (ULN) and 26 (44.8%) had at least one concentration ten times greater than ULN. Table 1 shows the clinical characteristics of the included patients. When a specific sample number (n) is not stated, in indicates that the data is complete. Sex disaggregated data is presented in Supplementary Tables S2 and S3. Characteristics of control patients for IHC and proteomics are described in Table 2 and their histopathological findings are presented in Supplementary Fig. S2.

**Table 1:** Clinical characteristics of included patients.

	Wild-type YF (n = 68)	YEL-AVD (n = 5)	Total YF (n = 73)	Proportion 95% CI
Demography				
Male sex^a^	59 (86.8%)	3 (60.0%)	62 (84.9%)	76.7%–93.1%
Female sex^a^	9 (13.2%)	2 (40.0%)	11 (15.1%)	6.9%–23.3%
Age (years-old)	49 (35.5–60)	37 (32–49)	48 (34–60)	
Previous medical condition				
Hypertension	19 (27.9%)	2 (40.0%)	21 (28.8%)	18.4%–39.2%
Diabetes	8 (11.8%)	0	8 (11.0%)	3.8%–18.1%
Heart disease	5 (7.4%)	0	5 (6.8%)	1.1%–16.6%
Asthma/COPD	4 (5.9%)	0	4 (5.5%)	0.3%–10.7%
Habits				
Alcoholism	36 (52.9%)	1 (20.0%)	37 (50.7%)	39.2%–62.2%
Smoking	26 (38.2%)	1 (20.0%)	27 (37.0%)	25.9%–48.1%
Illicit drug use	11 (16.2%)	0	11 (15.1%)	6.9%–36.3%
Interval (days)				
Symptoms—hospitalization	4 (3–6)	5 (5–5)	4 (3–6)	
Symptoms—death	9 (7–11)	8 (7–8)	9 (7–11)	
Hospitalization–death	5 (3–6)	3 (3–4)	5 (3–6)	
In-hospital events and interventions				
Shock/vasopressors use	68 (100%)	5 (100%)	73 (100%)	100%–100%
Inotropes use	3 (4.4%)	1 (20.0%)	4 (5.5%)	0.3%–10.7%
Supraventricular tachyarrhythmia	14 (20.6%)	1 (20.0%)	15 (20.5%)	11.3%–29.8%
Ventricular tachyarrhythmia^b^	1 (1.5%)	0	1 (1.4%)	0.0%–4.0%
Bradiarritmia^b^	5 (7.4%)	0	5 (6.8%)	1.1%–12.6%
Faget sign	8 (11.8%)	0	8 (11.0%)	3.8%–18.1%
Dialysis	58 (85.3%)	4 (80.0%)	62 (84.9%)	76.7%–93.1%
Mechanical ventilation	68 (100%)	5 (100%)	73 (100%)	100%–100%
Liver transplant	4 (5.9%)	0	4 (5.5%)	0.3%–10.7%
Secondary infection	53 (77.9%)	3 (60.0%)	56 (76.7%)	67.0%–86-4%
Vaccine status				
YF vaccine	9 (13.2%)	5 (100%)	14 (19.2%)	10.1%–28.2%
Vaccination > 10 days before symptoms	2 (2.9%)	0	2 (2.7%)	0.0%–6.5%
Electrocardiogram	n = 41 (60.3%)	n = 1 (20.0%)	n = 42 (57.5%)	
Non-sinus rhythm	18 (43.9%)	0	18 (42.9%)	27.9%–57.8%
Repolarization abnormalities	12 (29.3%)	0	12 (29.3%)	15.3%–43.2%
Sinus bradycardia	11 (26.9%)	0	11 (26.2%)	12.9%–39.5%
Branch block	8 (19.5%)	0	2 (4.8%)	0.0%–11.2%
Electrically inactive area	10 (24.4%)	0	10 (23.8%)	10.9%–36.7%
1st degree AV block	1 (2.4%)	0	1 (2.4%)	0.0%–7.0%
Normal	7 (17.1%)	1 (100%)	8 (19.0%)	7.2%–30.9%
Echocardiogram	n = 23 (33.8%)	n = 1 (20.0%)	n = 24 (32.9%)	
LV dilatation	2 (8.7%)	0	2 (8.3%)	0.0%–19.4%
EF < 50%	3 (13.0%)	0	3 (12.5%)	0.0%–25.7%
Segmental LV wall motion abnormalities	2 (8.7%)	0	2 (8.3%)	0.0%–19.4%
Troponin	n = 58 (85.3%)	n = 0	n = 58 (79.5%)	
Dosage > ULN	54 (93.1%)		54 (93.1%)	86.6%–99.6%
Dosage > 10x ULN	26 (44.8%)		26 (44.8%)	32.0%–57.6%
NT-proBNP	n = 8 (11.8%)	n = 0	n = 8 (11.0%)	
>900 pg/mL	4 (50.0%)		4 (50%)	15.4%–84.6%

CI: confidence interval; COPD: chronic obstructive pulmonary disease; Faget sign: relative bradycardia in the presence of fever; YF: yellow fever; YEL-AVD: yellow fever vaccine-associated viscerotropic disease; AV: atrioventricular; LV: left ventricle; EF: ejection fraction; ULN: upper limit of normal.

a Sex assigned at birth.

b Arrhythmias in the context of cardiac arrest were not reported.

**Table 2:** Clinical and demographic characteristics of control patients.

	Sex^a^	Age	Comorbidities	Cause of death
IHC CV controls (n = 4)	4 F	75 (64–82)	HTN (4); DM (4); dementia (1); COPD (1)	Acute pulmonary edema (4)
IHC Sepsis controls (n = 5)	3 M; 2 F	71 (21–85)	HTN (1); DM (1); CAD (1); breast cancer (1); chronic hepatic disease (1)	Pneumonia (3), peritonitis due to ischemic bowel necrosis (1), appendicitis (1)
Proteomics CV controls (n = 6)	3 M; 3 F	59 (51–71)	HTN (3); DM (1); metastatic cancer (1)	Acute pulmonary edema (1), acute aortic dissection (1), rupture of aortic aneurysm (1), pulmonary embolism (1), acute myocardial infarction (1), arrhythmia secondary electrolyte imbalance (1)
Proteomics sepsis controls (n = 6)	4 M; 2 F	47 (22–83)	HTN (2); DM (1); CAD (1); chronic hepatic disease (1)	Pneumonia (3), peritonitis due to ischemic bowel necrosis (2), appendicitis (1)

IHC: immunohistochemistry; CV: cardiovascular; F: female; M: male; HTN: hypertension; DM: diabetes mellitus; CAD: coronary artery disease; COPD: chronic obstructive pulmonary disease.

a Sex assigned at birth.

### Histopathology

The autopsies of patients with YF showed, in all cases, the typical liver alterations of the disease: midzonal hepatitis with steatotic and apoptotic hepatocytes, little associated inflammatory infiltrate, and viral antigens in degenerated hepatocytes and inflammatory cells observed through IHC. The other organs showed congestion and diffuse hemorrhage on macroscopy, with acute tubular necrosis and mesenteric ischemia being almost universal findings. The main immediate cause of death was refractory shock, due to sepsis and hemorrhages, mainly in the gastrointestinal and respiratory tracts.

**Fig. 1:**
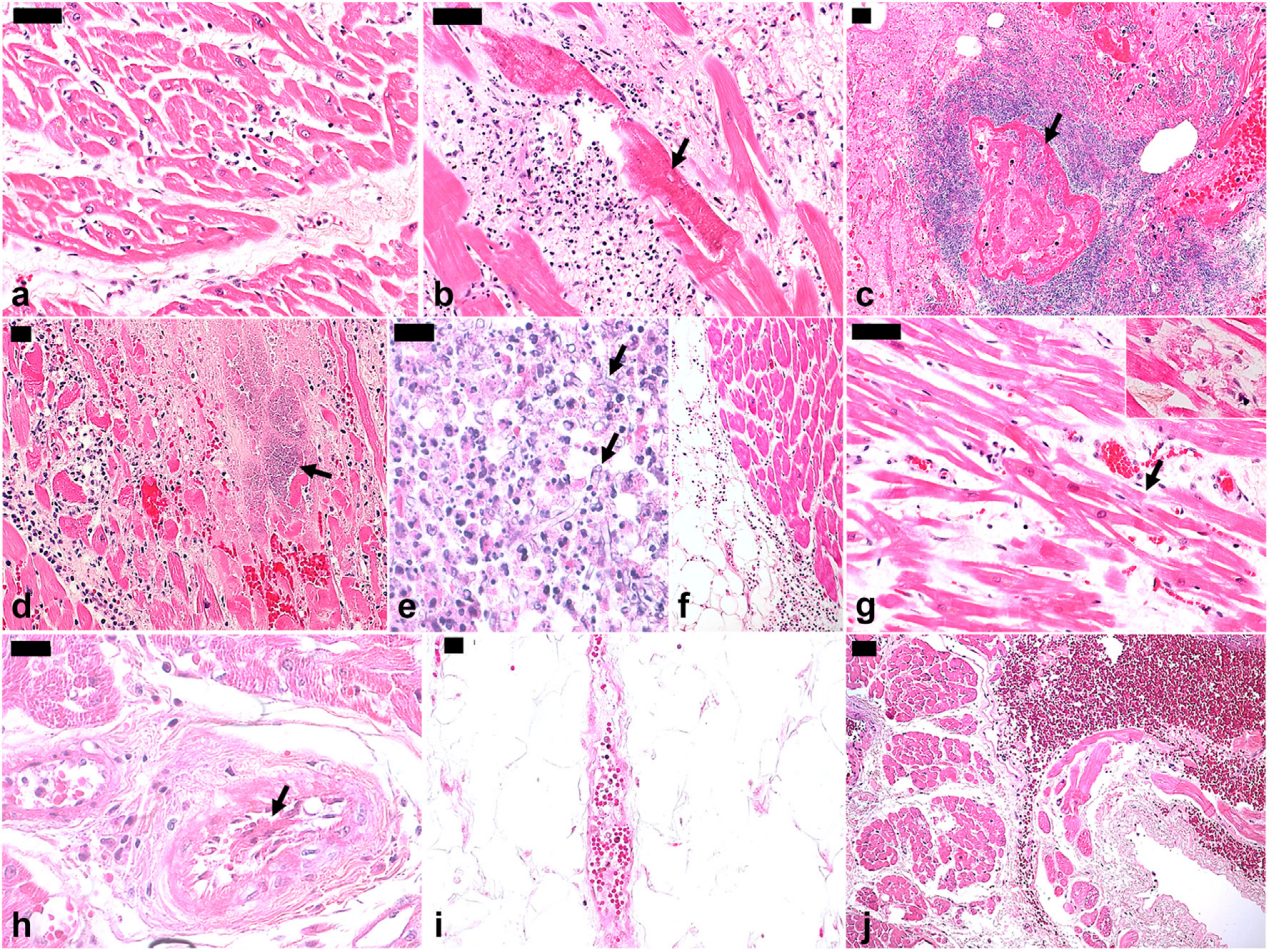
**Histopathological cardiac findings in autopsy cases of severe yellow fever.** a. Myocardial interstitial mononuclear inflammatory infiltrate and associated edema. b. Myocarditis, with mixed inflammatory infiltrate damaging cardiac fibers with dystrophic calcification (arrow). c. Secondary myocarditis due to Gram-negative bacilli, with hemorrhagic vascular necrosis (arrow). d. Myocarditis secondary to Gram-positive cocci colonies (arrow). e. Secondary myocarditis due to hyphomycetes with morphology compatible with *Aspergillus spp* (arrows). f. Epicarditis due to mixed inflammatory infiltrate. g. Hyaline necrosis, in a contraction band of cardiac fibers (arrow), fiber myolysis (inset), mononuclear inflammatory infiltrate and interstitial edema. h. Myocardial artery with tumefaction of endothelial cells, and fibrin within vascular lumen (arrow), perivascular mononuclear inflammatory infiltrate and edema. i. Epicardial venule with fibrinoid necrosis of the endothelium. j. Hemorrhage in the subendocardium and myocardium. Yellow fever subtype: a–g, i and j: wild-type; h: yellow fever vaccine-associated viscerotropic disease. Hematoxylin-Eosin. Magnification: C, F, J:100x; a, b, d, g, and i: 200x; e and h: 400x. Scale bars: 20 μm (c–e, h, i); 50 μm (a, b, g, j).

On macroscopy, 78.9% of the hearts had a weight bigger than the ULN (350 g). Sixty percent had increased LV wall thickness and 22.9% increased RV wall thickness. Aortic atherosclerosis (74.4%), coronary atherosclerosis (31.5%), and epicardial hemorrhages (31.5%) were frequently observed. In histology, the most frequent abnormalities were interstitial fibrosis (93.2%), cardiomyocyte hypertrophy (93.2%) and endothelial abnormalities (91.8%). We also observed cardiomyocyte necrosis (68.5%), suggestive of ischemic necrosis due to refractory shock, often associated with dystrophic calcification and contraction band necrosis, and interstitial hemorrhage (46.6%), especially in the subendocardium. Myocarditis was found in 14 (19.2%) patients, of which 5 had another microorganism involved, and 9 (13.2%) were considered viral myocarditis. Of those, 4 had mild focal inflammation, 4 had more severe inflammatory infiltrate in multiple foci and 1 had an abscess. Histopathological findings were not associated with prevalent comorbidities (hypertension and diabetes), except for hypertension and coronary atherosclerosis (p = 0.02, Fisher exact test). Fig. 1 shows images of histopathological findings.

**Fig. 2:**
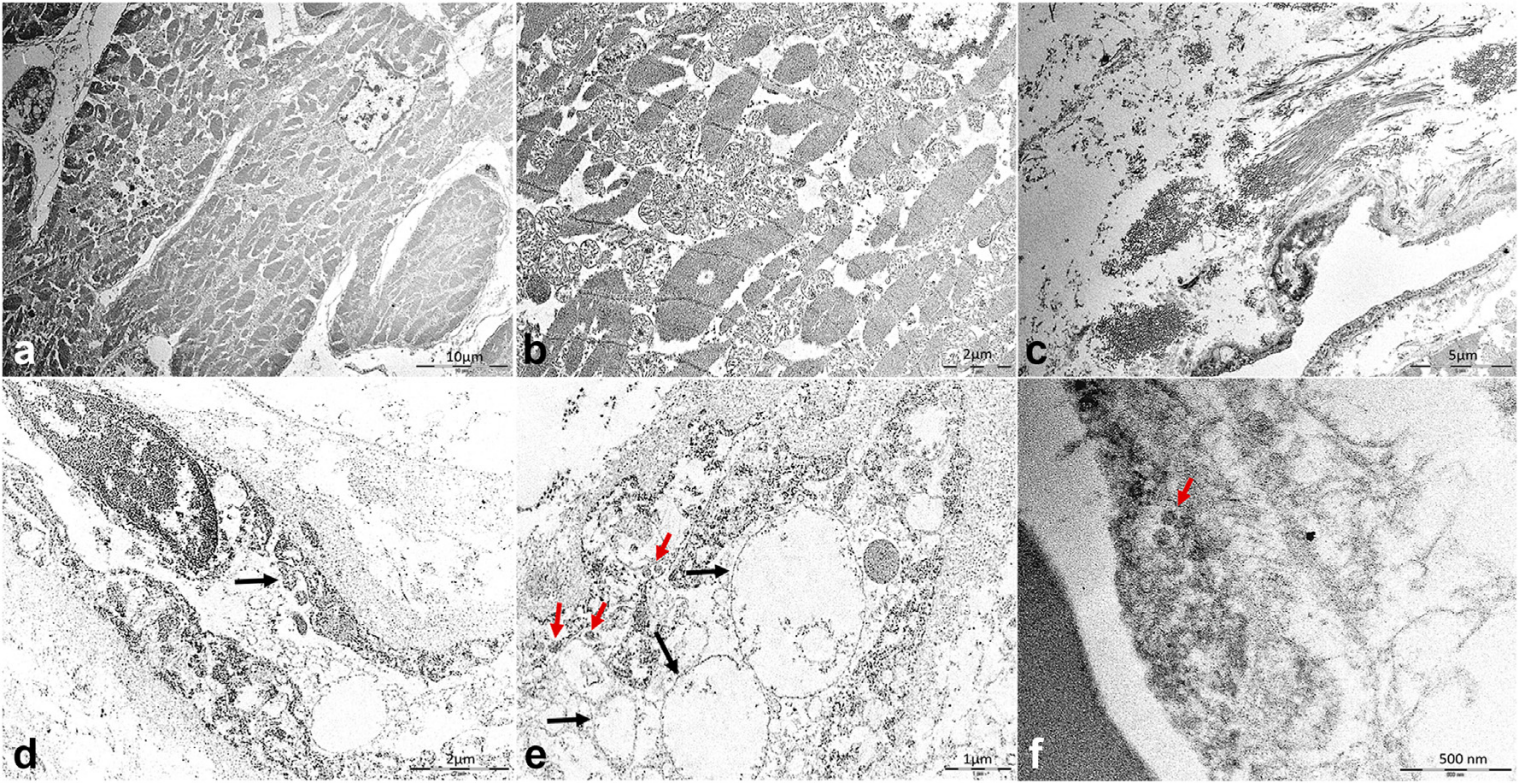
**Ultrastructural cardiac findings in autopsy cases of severe yellow fever.** a. Disarrangement of sarcomeres and mitochondrias in the cardiomyocytes. b. Mitochondriosis and rupture of mitochondrial cristae. c. Dissociated collagen fibers due to perivascular edema. d. Myocardial endothelial cells with swollen cytoplasm, displaying pseudopods (arrow). e. Cytoplasm of endothelial cells showing vesicular degeneration of the smooth endoplasmic reticulum (black arrows), with some vesicles showing round electron-dense virus-like particles, measuring 110–150 nm (red arrows). f. Endothelial cell cytoplasm showing endoplasmic reticulum vesicle, containing two electron-dense virus-like particles, measuring between 80 and 90 nm (arrow). All images are from wild-type yellow fever cases. Scale bars: 500 nm (f); 1 μm (e); 2 μm (b, d); 10 μm (a).

**Fig. 3:**
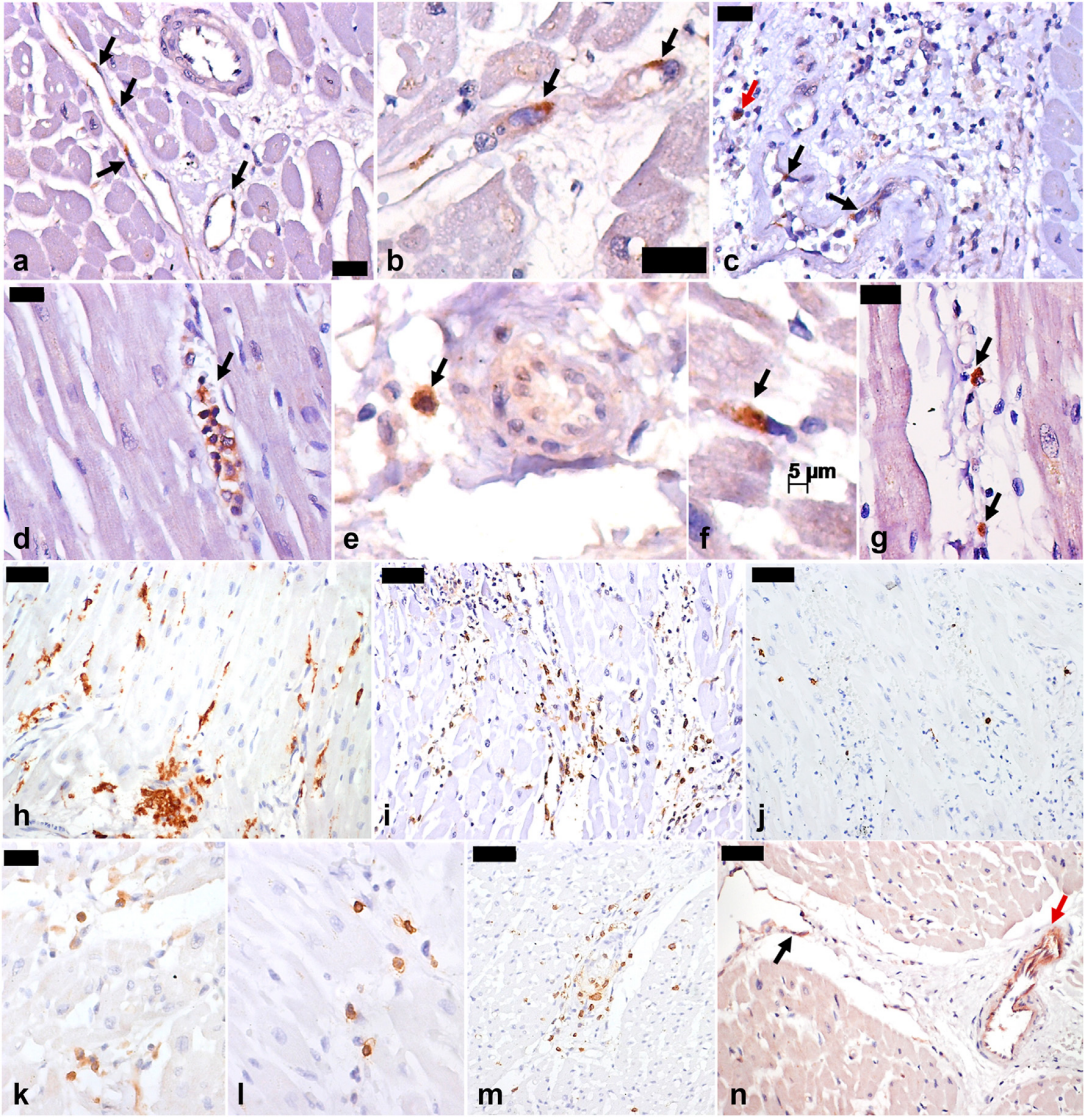
**Immunohistochemistry findings in myocardial tissue of autopsy cases of severe yellow fever.** a–g. Immunostaining of yellow fever virus antigen: within the cytoplasm of endothelial cells from myocardial vessels (a and b, arrows); within inflammatory cells (c, red arrow) and endothelial cells (c, black arrow) in focal mixed myocarditis, attributed to yellow fever; in circulating leukocytes, in a case with detectable YFV-RNA in the blood on the day of death (d, arrow); in perivascular inflammatory cells in the epicardium (e, arrow); in inflammatory cells among cardiac fibers (f and g, arrows). h–m. Myocardial inflammatory cell immunophenotype: CD68+ cells (h); CD45+ cells (i); NK (j) cells; T-CD4+ cells (k); T-CD8+ cells in a wild-type yellow fever case (l); and T-CD8+ cells in a case with acute viscerotropic disease associated with yellow fever vaccine and myocarditis (m). n. Increased angiopoietin-2 expression in venous (black arrow) and arterial (red arrow) vessels in cases of severe yellow fever. Yellow fever subtype: a–l, n: wild-type; m: yellow fever vaccine-associated viscerotropic disease. Peroxidase. Magnification: h, i, j, m, n: 100x; a, c, d, g, k, l: 200x; b, e and f: 400x. Scale bars: 5 μm (f); 20 μm (a–d, g, k); 50 μm (h–j, m, n).

The ultrastructural analysis found endothelial cell abnormalities, with cytoplasm swelling, pseudopods and vesicular degeneration of the endoplasmic reticulum. Fig. 2 shows images of ultrastructural findings. In one patient with numerous myocardial vessels positive for YFV-antigen (Fig. 3a and b), we identified round electron-dense particles, similar to flaviviruses virions, measuring between 80 and 150 nm, inside vesicles in the endoplasmic reticulum. We also found sarcomeric disarrangement, mitochondriosis, and rupture of mitochondrial cristae in cardiomyocytes, as well as perivascular interstitial edema.

### Immunohistochemistry

We performed IHC reactions in 24 (32.9%) patients; all of them had positive YFV-antigen in myocardial tissue, while 5 individuals had greater amounts of antigen (positive 2+). The most frequent spot of YFV-antigen staining was the cytoplasm of endothelial and inflammatory cells; we did not find YFV-antigen in cardiomyocytes. IHC also showed increased inflammatory cells in the myocardium, predominantly macrophages, with a median count of 14.3 CD68+ cells per field. Compared to controls, there was a statistically significant difference in CD45+ and CD68+ counts (Kruskal–Wallis test), with higher levels in the YF group. Among cases with myocarditis, there was interstitial inflammatory infiltration composed of macrophages and lymphocytes. We assessed the presence of angiopoietin-2 in 19 (26.0%) YF cases, 10 (52.6%) were classified as 3+, 9 (47.4%) as 2+, and 1 (5.3%) as 1+. There was a statistical difference compared to cardiovascular and sepsis controls (p = 0.007, Fisher exact test). Fig. 3 shows images of IHC findings.

### Viral RNA detection

RTq-PCR was performed in the myocardial tissue of 69 (94.5%) patients and tested positive in 66 (95.7%). Pathological, IHC and molecular analyses are described in Table 3.

**Table 3:** Histopathologic. Immunohistochemistry and molecular biology findings in included patients.

	Wild-type YF (n = 68)	YEL-AVD (n = 5)	Total YF (n = 73)	Proportion 95% CI
Heart weight (g)	434 (374.5–469.5)	318 (314–440)	424.3 (±92.7)	
Weight > 350 g	54/66 (81.8%)	2 (40.0%)	56/71 (78.9%)	69.4%–88.4%
LV thickness > 1.5 cm	40/65 (61.5%)	2 (40.0%)	42/70 (60.0%)	48.5%–71.5%
Interstitial fibrosis	64 (64.1%)	4 (80.0%)	68 (93.2%)	87.4%–98.9%
Perivascular	31 (45.6%)	2 (40.0%)	33 (45.2%)	33.8%–56.6%
Multifocal	22 (32.4%)	1 (20.0%)	23 (34.5%)	20.9%–42.2%
Diffuse	11 (16.2%)	1 (20.0%)	12 (16.4%)	7.9%–24.9%
Cardiomyocites hypertrophy	64 (64.1%)	4 (80.0%)	68 (93.2%)	87.4%–98.9%
Endothelial abnormalities	62 (91.2%)	5 (100%)	67 (91.8%)	85.5%–98.1%
Mild	49 (72.1%)	2 (40.0%)	51 (69.9%)	59.3%–80.4%
Moderate	10 (14.7%)	2 (40.0%)	12 (16.4%)	7.9%–24.9%
Severe	3 (4.4%)	1 (20.0%)	4 (5.5%)	0.3%–10.7%
Fiber necrosis	45 (66.2%)	5 (100%)	50 (68.5%)	57.8%–79.1%
Coronary atherosclerosis	35 (51.5%)	3 (60.0%)	38 (52.1%)	40.6%–63.5%
Interstitial hemorrhage	32 (47.1%)	2 (40.0%)	34 (46.6%)	35.1%–58.0%
Epicarditis	23 (33.8%)	4 (80.0%)	27 (37.0%)	25.9%–48.1%
Myocarditis	10 (14.7%)	4 (80.0%)	14 (19.2%)	10.1%–28.2%
Secondary	5 (7.4%)	0	5 (6.8%)	1.1%–16.6%
Bacterium	3 (4.4%)	0	3 (4.1%)	0.0%–8.7%
Fungus	1 (1.5%)	0	1 (1.4%)	0.0%–4.0%
Chagas	1 (1.5%)	0	1 (1.4%)	0.0%–4.0%
Viral	5 (7.4%)	4 (80.0%)	9 (13.2%)	4.8%–19.9%
Focal	2 (2.9%)	2 (40.0%)	4 (5.5%)	0.3%–10.7%
Multifocal	2 (2.9%)	2 (40.0%)	4 (5.5%)	0.3%–10.7%
Abscess	1 (1.5%)	0	1 (1.4%)	0.0%–4.0%
Immunohistochemistry (mean count per HPF)	n = 19 (27.9%)	n = 5 (100%)	n = 24 (32.9%)	
CD68+ cells	13.2 (9.8–18.7)	18.3 (18.2–25.5)	14.3 (10.6–19.5)	
CD45+ cells	2.6 (2.1–4.2)	6.8 (3.3–10.6)	2.8 (2.3–5.4)	
CD57+ cells	0.3 (0.1–0.4)	0.3 (0.1–0.6)	0.3 (0.1–0.5)	
CD4+ cells	0.8 (0.5–1.1)	4.5 (0.6–4.6)	0.8 (0.5–1.5)	
CD8+ cells	0.8 (0.4–1.5)	2.4 (2.3–8.5)	1.0 (0.4–2.3)	
Positive YF viral antigen	19 (100%)	5 (100%)	24 (100%)	100%–100%
Positive 1+	15 (78.9%)	4 (80.0%)	19 (79.2%)	62.9%–95.4%
Positive 2+	4 (21.1%)	1 (20.0%)	5 (20.8%)	4.6%–37.1%
RT-qPCR for YF	n = 64 (87.7%)	n = 5 (100%)	n = 69 (94.5%)	
Positive	61 (95.3%)	5 (100%)	66 (95.7%)	90.8%–100%
Cardiac conduction system	n = 7 (10.3%)	n = 1 (20.0%)	n = 8 (11.0%)	
Edema, hemorrhages and inflammatory infiltrate	7 (100%)	1 (100%)	8 (100%)	100%–100%
SN artery fibrinoid necrosis	2 (28.6%)	0	2 (25.0%)	0%–55.0%
SN fibrosis	1 (14.3%)	1 (100%)	2 (25.0%)	0%–55.0%
Conduction system fiber necrosis	1 (14.3%)	0	1 (12.5%)	0%–35.4%
Mycotic thrombus	1 (14.3%)	0	1 (12.5%)	0%–35.4%
Positive YF viral antigen	3 (42.9%)	1 (100%)	4 (50.0%)	15.4%–84.6%

CI: confidence interval; LV: left ventricle; HPF: high-power field; CD: cluster of differentiation; YF: yellow fever; RT-qPCR: reverse transcriptase quantitative polymerase chain reaction; YEL-AVD: yellow fever-associated viscerotropic disease; SN sinus node. The patient with associated Chagas’ disease was not included in the analysis of the conduction system.

### Cardiac conduction system

**Fig. 4:**
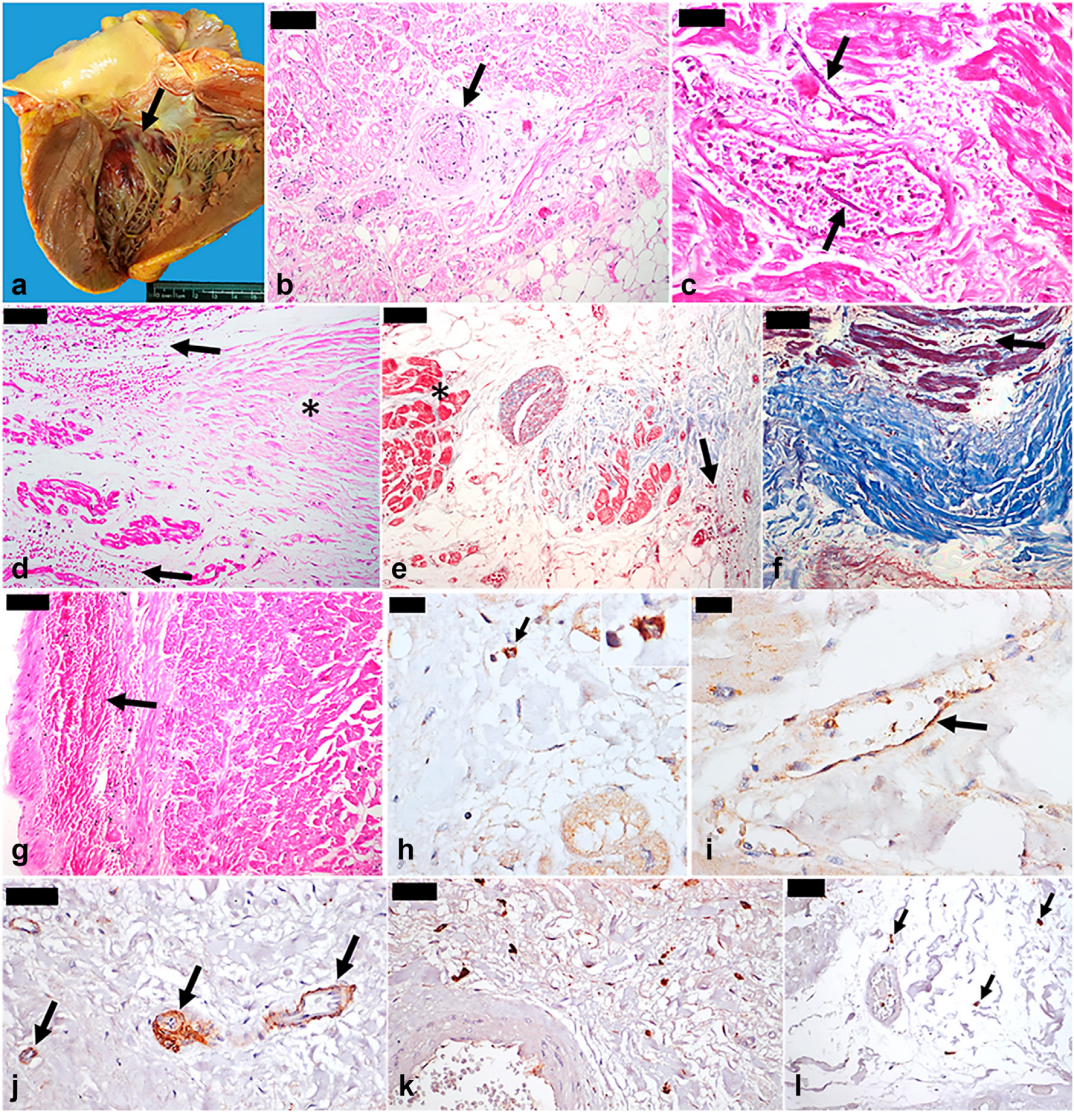
**Pathology findings of the cardiac conduction system in autopsy cases of severe yellow fever.** a. Macroscopy of the heart demonstrates a common finding in cases of severe yellow fever: subendocardial hemorrhage on the basal left part of the interventricular septum, in the topography of the conduction system (left bundle branch). b. Sinoatrial nodal artery (arrow) with endothelial swelling, fibrin aggregates within vascular lumen, edema of the vessel wall, and perivascular interstitial edema. c. Artery close to the sinoatrial node showing mycotic thrombus (arrows), in case of secondary infection by *Aspergillus* spp. d. Edema and interstitial hemorrhage (arrows) around the compact node (asterisk). e. Interstitial edema and hemorrhage (arrow) around the bundle of His (asterisk). f. Edema and interstitial hemorrhage (arrow) around the proximal portion of the left bundle branch. g. Edema and interstitial hemorrhage (arrow) around the distal portion of the left bundle branch. h and i. Immunostaining of yellow fever virus antigen in the cytoplasm of interstitial macrophages close to the sinoatrial node (h, arrow and inset) and within endothelial cells of some vessels in the myocardium (i, arrow), close to the bundle of His and left bundle branch. j. Increased angiopoietin-2 expression in vessels close to the sinoatrial node. k. Inflammatory infiltrate by activated macrophages (CD68+) around the sinoatrial nodal artery. l. Immunostaining for CD45 in interstitial lymphocytes (arrows), close to the bundle of His. All images are from wild-type yellow fever cases. Hematoxylin-Eosin: b–d, g; Masson’s trichrome: e, f; Peroxidase: h–l. Magnification: b, d–g: 100x; c, j–l: 200x; h, i: 400x. Scale bars: 20 μm (h, i); 50 μm (c, j–l); 100 μm (b, d–f, g).

The microscopy of the conduction system from 8 (11.0%) patients showed edema, hemorrhages, and mild macrophagic inflammatory infiltrate in all cases; fibrinoid necrosis in the sinoatrial nodal artery in 2 (25.0%) patients; sinoatrial node fibrosis in 2 (25%); conduction system fibers necrosis in 1 (12.5%), and a mycotic thrombus by hyphomycetes in 1 (12.5%). We also found YFV-antigen in 4 (50.0%) patients. The patient with associated Chagas’ disease was not included in the analysis of the conduction system. Fig. 4 shows images of the cardiac conduction system pathology.

### Proteomics

**Fig. 5:**
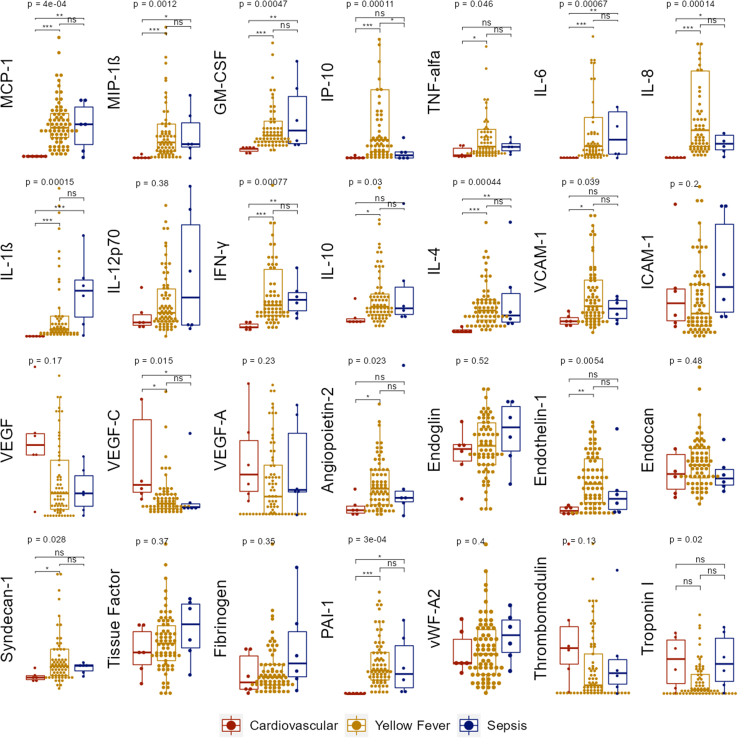
**Tissue proteomics analysis of cytokines and endothelial biomarkers.** Cytokines and endothelial lesion biomarkers dosages performed through multiplex immunoassay on fragments of frozen myocardial tissue obtained from autopsy from 69 patients with yellow fever, 6 with cardiovascular death and 6 with sepsis. All biomarkers dosages were normalized by total protein of each sample and only comparative data are shown. The Kruskal–Wallis test was used for comparisons between the 3 groups. When there was statistical significance, Dunn’s post-hoc test with Benjamini-Hochberg correction was used for multiple comparisons. ∗p < 0.05; ∗∗p < 0.01; ∗∗∗p < 0.001; ∗∗∗∗p < 0.0001; ns: not significant. TNF: tumor necrosis factor; IFN-γ: Interferon-gama: interferon, IL: interleukin; MCP-1: monocyte chemoattractant protein 1; MIP1-β: macrophage inflammatory protein 1–beta; IP-10: Interferon gamma-induced protein 10; GM-CSF: granulocyte and macrophage colony stimulating factor; VEGF: vascular endothelial growth factor; VCAM-1: vascular cell adhesion molecule 1; ICAM-1: Intercellular adhesion molecule 1; PAI-1: plasminogen activation inhibitor; vWF-A2: von Willebrand factor A2.

Samples from 69 (94.5%) patients with YF, including all the patients with YEL-AVD, and 12 controls, of which 6 died from sepsis and 6 from cardiovascular disease, underwent protein multiplex immunoassay. In immune-related proteins analysis, we found higher concentrations of all cytokines but IL12p70 in the YF group compared to the cardiovascular group. There was no statistically significant difference in concentrations of cytokines between the YF and the sepsis group, except for IP-10, which was higher in YF patients than all control patients (p < 0.001 vs cardiovascular; p = 0.02 vs sepsis; Dunn test). Among endothelial proteins, the YF group showed higher levels of angiopoietin-2, endothelin-1, syndecan-1, VCAM-1 and PAI-1 compared to the cardiovascular group and lower levels of VEGF-C; there were no statistically significant differences between the sepsis and the YF patients. Group comparisons are shown in Fig. 5.

### YEL-AVD subanalysis

We performed a subanalysis of the five patients with YEL-AVD. They all took the YF vaccine within five days or less from the beginning of the symptoms. We found 4 (80%) cases with viral myocarditis; two had focal inflammatory foci and two had multifocal myocarditis. In all cases, we found endothelial abnormalities and fiber necrosis; interstitial fibrosis and hypertrophy were described in 4 (80%). One patient had an acute thrombus in an intramyocardial artery. On comparison with wild-type YF patients, those with YEL-AVD showed higher levels of mean CD8+ count per high-power field (2.4 vs 0.8; p = 0.03; Wilcoxon rank-sum test) and higher levels of IFN-γ (p = 0.03; Wilcoxon rank-sum test) on proteomics analysis (Supplementary Fig. S3); there was no statistically significant difference in the remaining IHC and proteomics analyses.

## Discussion

This study describes the pathology of the hearts of 73 patients who died of YF in Brazil during the world’s largest YF epidemics of the 21st century. We demonstrated a high prevalence of cardiac abnormalities in those patients. YF-associated myocardial injury clinically manifested as bradycardia, arrhythmias, elevated troponin and shock. Pathological analysis showed endothelial abnormalities, myocardial fibrosis, hypertrophy, cardiomyocyte necrosis, and mononuclear myocarditis. We also detected YFV components in myocardial tissue: YFV-antigens in endothelial and inflammatory cells by IHC, YFV RNA through RT-qPCR, and virus-like particles that may correspond to YF virions in endothelial cells via EM. Protein multiplex analysis showed high levels of cytokines and endothelial lesion biomarkers in myocardial tissue.

Cardiovascular complications are frequent in severe infections and have been described in sepsis and multiple viral diseases, such as COVID-19 and arboviral infections, including dengue fever, Zika, and chikungunya, with reports of viral myocarditis.^22^, ^23^, ^24^, ^25^, ^26^ YF is considered a systemic disease and, despite the liver being the most affected organ, previous works have demonstrated evidence of cardiac involvement.^6^, ^7^, ^8^, ^9^, ^10^ We confirmed this hypothesis and explored its mechanisms from a pathology perspective. We did not find YFV-antigens or viral particles by IHC or EM, respectively, within cardiomyocytes, suggesting that YFV does not have direct tropism for the cardiac cell, unlike recent evidence in an experimental model of infection by the Zika virus, another endemic arbovirus in the Brazil.^27^ Particularities of the various arboviruses, in terms of tropism and host-pathogen interaction, may explain these differences and future studies may elucidate this issue.

Inflammation may be central to the YF-associated myocardial injury, as it is to other infectious diseases. We found increased inflammatory cellular infiltrate in myocardium; in most cases, the cells were predominantly located in the perivascular interstitium. The immunostaining showed a preponderance of macrophages, which have been previously associated with myocardial injury in both sepsis and COVID-19.^28^^,^^29^ Moreover, high levels of multiple inflammatory cytokines are present in myocardial tissue of our YF-cases. They were detected at greater levels than in patients who died from cardiovascular disease and had a similar profile to septic patients. Elevated concentrations of many cytokines, such as IL-1β, TNF-α and IL-6 are linked to myocardial dysfunction in septic patients.^26^^,^^30^ We found a statistically significant difference between YF and sepsis in IP-10 levels. IP-10, also known as CXCL-10, is a chemokine involved in the recruitment of lymphocytes, macrophages and NK cells. High levels of this cytokine have been previously described in patients with viral diseases, such as COVID-19, dengue fever and Zika, and have been associated with worse prognosis.^31^, ^32^, ^33^ Further studies may explore the role of IP-10 in severe YF. Such a proinflammatory local environment may be secondary to a systemic cytokine storm. Both cellular and humoral components of hyperinflammation may likely be involved in the pathophysiology of YF-associated myocardial injury.

We found viral myocarditis in 9 (13.2%) patients. Of these, 4 had focal inflammation, 4 had multifocal myocarditis, and 1 had an abscess. These patients predominantly displayed a mononuclear infiltrate. There had been only a few previous reports in the literature of myocarditis caused by the YFV.^6^^,^^12^, ^13^, ^14^ None of our patients had a clinical diagnosis of myocarditis. This could be explained by several factors, such as the difficulty of diagnosing it without histopathology, especially considering that the patients were clinically unstable to undergo cardiac magnetic resonance, and the great range of disease severity, from mild myocarditis to fulminant ones. It was not possible to determine the clinical relevance of myocarditis in this scenario, but it is plausible that it contributed to the shock and fatal outcome observed in all the patients. Myocarditis and myocardial hemorrhage due to the YFV-17DD strain found in the cases described here are similar, from the histological point of view, to previous reports made by our group in cases of YEL-AVD.^17^^,^^34^ These results corroborate that the vaccine strain can mediate not only severe adverse lesions in the liver and brain, but also in the heart, and, therefore, we suggest awareness regarding cardiovascular symptoms after administration of the 17DD vaccine. Clinical and pathological studies (human and experimental) can deepen this important matter.

Regarding the important endothelial lesion found in our cases, it is a hallmark in the pathogenesis of arboviral diseases, especially dengue fever, leading to capillary leakage and consequent distributive shock.^35^ Our study showed frequent endothelial abnormalities in histological analysis, with endothelial swelling in many cases and fibrinoid necrosis in some. In the proteomics analysis, we identified elevated biomarkers associated with endothelial damage, namely angiopoietin-2, endothelin-1, syndecan-1, VCAM-1 and PAI-1. Angiopoietin-2 is a vascular growth factor associated with capillary leakage in sepsis, and higher concentrations of this biomarker have been associated with organ dysfunction and worse prognosis.^36^ Elevated levels of endothelin-1 contribute to inflammatory activation and increased production of IL-1, TNF-α and IL-6; while higher syndecan-1, endothelin-1, and VCAM-1 are associated with worse prognosis in sepsis.^36^, ^37^, ^38^, ^39^ PAI-1 is a fibrinolysis inhibitor classically linked to disseminated intravascular coagulation, and may be involved in the YF coagulopathy, with high prevalence of hemorrhages. Yet, contrary to other infectious diseases, arterial thrombosis and microthrombi were rarely seen. Noteworthy, we also detected YFV-antigens by IHC, as well as virus-like particles, via EM, within endothelial cells. These findings support the hypothesis that direct YFV invasion is a key mechanism of endothelial damage in severe YF. The interaction of the NS1 protein with the glycocalyx and angiopoietin-2, analogous to what is seen in dengue fever, may be an explanation.^40^ However, further studies are required to confirm this hypothesis.

Bradycardia is a frequent finding in YF cohorts, corresponding to the classical Faget’s sign.^6^^,^^7^ In our cases, ECGs also often showed sinus bradycardia, along with other electrical abnormalities like non-sinus rhythm and anomalous repolarization. Identifying a histopathological background that justifies arrhythmias, including sudden death, is challenging, as seen in autopsies from patients who died from channelopathies.^41^ We represented histologically the conduction system of 8 YF-patients and observed edema and hemorrhages in nodes and branches. We also found fibrinoid necrosis of the sinoatrial node artery in two patients. There were inflammatory infiltrate and YFV-antigens by IHC in the cardiac conduction system. These findings may provide pathological evidence to explain the occurrence of bradycardia and arrhythmias in severe YF, whose causative mechanisms are likely more complex, involving multiple factors, systemic and in situ inflammation, and endothelial damage.

The present findings have a potential clinical impact. The high prevalence of cardiac abnormalities could encourage physicians to give particular attention to the cardiovascular system in patients with severe YF through continuous monitoring and complementary exams. This may lead to earlier and more accurate supportive measures. Potential mechanisms of cardiac injury described in this study may be further explored in experimental research and may lead to the discovery of novel biomarkers and therapeutic targets. Furthermore, our results corroborate previous clinical studies on severe YF from the 2017–2019 epidemic in São Paulo, Brazil, published by our institutions.^8^^,^^42^ Most of the fatal cases described in these studies were included in the present work. Ho et al. described that, among 79 cases, 35% YF cases had bradyarrhythmia on hospital admission and 27% developed tachyarrhythmias, associated with increased serum troponin. Our myocardial and conduction system analyses show some pathological substrate for this clinical observation. In the study by Kallas et al., neutrophil counts of 4000 cells per mL or greater on admission were independently associated with death.^42^ Our autopsy results show not only direct YFV-mediated lesions in target organs, such as the liver and heart, but also secondary bacterial or fungal infections, with systemic repercussions. The inflammatory profile of cardiac injury and the biomarkers of endothelial injury in YF severe cases are similar to control sepsis cases and endorse the findings of Kallas et al., with high neutrophil counts on admission being an initial marker for the occurrence of secondary sepsis.^42^ We believe that in addition to the three classic phases of yellow fever–the viremic phase, the remission of symptoms and the toxic phase, there is a fourth phase, the “secondary sepsis phase”, which strongly contributes to determining the patient’s prognosis.

There are some limitations in this study. Clinical data was collected retrospectively from medical records, which may have inaccurate or missing information. Exams such as ECG, echocardiogram, and troponin were performed in only a fraction of the patients’ sample, which may overestimate the real prevalence of cardiac abnormalities in YF, considering that attending physicians ordered the exams for those with higher pre-test probabilities. Autopsy studies describe a picture at the end of a pathological process, making it possible that part of our findings is a consequence of previous comorbidities, secondary infection (e.g. aspiration pneumonia, intestinal translocation, nosocomial fungal infections) and refractory shock. However, we still found evidence of direct action of YFV on tissues by modern investigative techniques in anatomical pathology. We did not validate our EM results with immunogold or with YFV-infected cultured cells due to technical conditions and this finding needs to be interpreted with parsimony. However, the case with virus-like particles examined under EM showed positive immunostaining for YFV-antigens in various myocardial vessels, had detectable YFV-RNA through RT-PCR, the virus-like particles found were round and located within dilated endoplasmic vesicles, and, finally, the tissue ultrastructural morphology can be affected by post-mortem artifacts.^43^ Another limitation is that our cases were infected with specific YFV strain in a particular geographical area, thus our data might not be generalizable to all YF endemic areas.

This study provides a rare opportunity to study the heart in an old tropical neglected disease. There are more than 30,000 deaths worldwide attributed to YF every year. Still, there is little interest and funding for research.^2^^,^^44^ Outbreaks and epidemics outside the endemic areas highlight the risk for reemergence of the disease in several parts of the world, and reinforce the need for a coordinated global action, including research promotion.

In conclusion, we have described characteristics of YF-associated myocardial injury in autopsies of patients from recent epidemics in Brazil. There are frequent clinical and pathological abnormalities, with evidence of viral presence in myocardial tissue by RT-PCR, IHC and possibly by EM. Both local and systemic mechanisms are involved, such as mononuclear myocarditis, diffuse endothelial injury, and high levels of vascular biomarkers and inflammatory cytokines in situ. IP-10 is remarkably elevated and may play an important role in YF-associated injury. These findings may impact the clinical care of patients with severe YF and provide a path for future studies to explore the pathogenic mechanisms described herein.

## Contributors

FRG–writing (original draft), formal analysis, visualization. VDA–writing (review & editing), investigation, validation. CSF–writing (review & editing), investigation. SZP–writing (review & editing), investigation, formal analysis. MPC–writing (review & editing), investigation, formal analysis. MVG–writing (review & editing), investigation. HTP–writing (review & editing), investigation. FLL–writing (review & editing), investigation. CEM–writing (review & editing), investigation. HYL–writing (review & editing), resources. JS–writing (review & editing), resources. SMF–writing (review & editing), investigation. LFFS–writing (review & editing), resources. TM–writing (review & editing), supervision. VAFA–writing (review & editing), supervision, resources, funding acquisition. PHNS–writing (review & editing), supervision, funding acquisition. LA–writing (review & editing), methodology. MD-writing (review & editing), supervision, funding acquisition. ANDN–writing (original draft), conceptualisation, data curation, visualization, formal analysis, methodology, project administration. Both FRG and ANDN have directly accessed and verified the underlying data reported in the manuscript. All authors read and approved the final version of the manuscript.

## Data sharing statement

The data that support the findings of this study are available with publication, from the corresponding author, ANDN, upon reasonable request.

## Declaration of interests

The authors declare no conflict of interests.
