# Supplementary material for: Understanding yellow fever-associated myocardial injury: an autopsy study
Source: eBioMedicine. 2023 Sep 25;96:104810. doi: 10.1016/j.ebiom.2023.104810 (PMC10550587; doi:10.1016/j.ebiom.2023.104810)
Supplement: Supplementary Figs. S1–S3 and Tables S1–S3 [file mmc1.docx]

Supplementary Table 1 – Primers and probe used in RT-qPCR for Yellow Fever Virus

| **Nucleic acid** | **Sequence** |
| --- | --- |
| Reverse primer | GTCRRTTCTCTGCTAATCGCTCA |
| Forward primer | ATTGAGGTGYATTGGTCTGC |
| Probe | FAM-AGTTGCTAGGCAATAAA-BBQ |

Supplementary Table 2 – Clinical characteristics of included patients stratified by sex

|  | **Female sex ^a^ (n=11)** | **Male sex ^a^ (n=62)** | **Total (n=73)** |
| --- | --- | --- | --- |
| **Age (years-old)** | 48 (32-70) | 48 (36-58) | 48 (34-60) |
| **Previous medical condition** |  |  |  |
| Hypertension | 5 (45·5%) | 16 (25·8%) | 21 (28·8%) |
| Diabetes | 3 (27·3%) | 5 (8·1%) | 8 (11·0%) |
| Heart disease | 0 | 5 (8·1%) | 5 (6·8%) |
| Asthma / COPD | 0 | 4 (6·5%) | 4 (5·5%) |
| **Habits** |  |  |  |
| Alcoholism | 0 | 37 (59·7%) | 37 (50·7%) |
| Smoking | 2 (18·2%) | 25 (40·3%) | 27 (37·0%) |
| Illicit drug use | 0 | 11 (177%) | 11 (15·1%) |
| **Interval (days)** |  |  |  |
| Symptoms – hospitalization | 5 (4·5-6·0) | 4 (3·0-5·3) | 4 (3-6) |
| Symptoms – death | 8 (7·0-13·5) | 9 (7·0-10·8) | 9 (7-11) |
| Hospitalization - death | 3 (2·0-5·5) | 5 (3·0-6·0) | 5 (3-6) |
| **In-hospital events and interventions** |  |  |  |
| Shock / vasopressors use | 11 (100%) | 62 (100%) | 73 (100%) |
| Inotropes use | 1 (9·1%) | 3 (4·8%) | 4 (5·5%) |
| Supraventricular tachyarrhythmia | 1 (9·1%) | 14 (22·6%) | 15 (20·5%) |
| Ventricular tachyarrhythmia ^b^ | 0 | 1 (1·6%) | 1 (1·4%) |
| Bradiarritmia ^b^ | 1 (9·1%) | 4 (6·5%) | 5 (6·8%) |
| Faget sign | 0 | 8 (12·9%) | 8 (11·0%) |
| Dialysis | 6 (54·5%) | 56 (90·3%) | 62 (84·9%) |
| Mechanical ventilation | 11 (100%) | 62 (100%) | 73 (100%) |
| Liver transplant | 0 | 4 (6·5%) | 4 (5·5%) |
| Secondary infection | 7 (63·6%) | 49 (79·0%) | 56 (76·7%) |
| **Vaccine status** |  |  |  |
| YF vaccine | 5 (45·5%) | 9 (14·5%) | 14 (19·2%) |
| Vaccination > 10 days before symptoms | 1 (9·1%) | 1 (1·6%) | 2 (2·7%) |
| **Electrocardiogram** | n=5 | n=37 | n=42 |
| Non-sinus rhythm | 0 | 18 (48·6%) | 18 (42·9%) |
| Repolarization abnormalities | 0 | 12 (34·2%) | 12 (29·3%) |
| Sinus bradycardia | 0 | 11 (29·7%) | 11 (26·2%) |
| Branch block | 0 | 2 (5·4%) | 2 (4·8%) |
| Electrically inactive area | 1 (20·0%) | 9 (24·3%) | 10 (23·8%) |
| 1st degree AV block | 0 | 1 (3·4%) | 1 (2·4%) |
| Normal | 2 (40·0%) | 6 (16·2%) | 8 (19·0%) |
| **Echocardiogram** | n=2 | n=22 | n=24 |
| LV dilatation | 0 | 2 (9·1%) | 2 (8·3%) |
| EF < 50% | 0 | 3 (13·6%) | 3 (12·5%) |
| Segmental LV wall motion abnormalities | 0 | 2 (9·1%) | 2 (8·3%) |
| **Troponin** | n=7 | n=51 | n=58 |
| Dosage > ULN | 7 (100%) | 47 (92·2%) | 54 (93·1%) |
| Dosage > 10x ULN | 2 (28·6%) | 24 (47·1%) | 26 (44·8%) |
| **NT-proBNP** | n=0 | n=8 | n=8 |
| >900 pg/mL |  | 4 (50·0%) | 4 (50·0%) |

^a^ Sex assigned at birth. ^b^ arrhythmias in the context of cardiac arrest were not reported. CI: confidence interval; COPD: chronic obstructive pulmonary disease; Faget sign: relative bradycardia in the presence of fever; YF: yellow fever; YEL-AVD: yellow fever vaccine-associated viscerotropic disease; AV: atrioventricular; LV: left ventricle; EF: ejection fraction; ULN: upper limit of normal

Table 3 - Histopathologic. immunohistochemistry and molecular biology findings in included patients stratified by sex

|  | **Female sex ^a^ (n=11)** | **Male sex ^a^ (n=62)** | **Total (n=73)** |
| --- | --- | --- | --- |
| Heart weight (g) | 322·0 (293·0-388·5) | 441·0 (393·8-471·0) | 424·3 (±92·7) |
| Weight > 350 g | 4 (36·4%) | 52 / 60 (86·7%) | 56 / 71 (78·9%) |
| LV thickness > 1·5 cm | 1 (9·1%) | 41 / 59 (69·5%) | 42 / 70 (60·0%) |
| Interstitial fibrosis | 8 (72·7%) | 60 (96·8%) | 68 (93·2%) |
| Perivascular | 5 (45·5%) | 28 (45·2%) | 33 (45·2%) |
| Multifocal | 2 (18·2%) | 21 (33·9%) | 23 (34·5%) |
| Diffuse | 1 (9·1%) | 11 (17·7%) | 12 (16·4%) |
| Cardiomyocites hypertrophy | 8 (72·7%) | 60 (96·8%) | 68 (93·2%) |
| Endothelial abnormalities | 10 (90·9%) | 57 (91·9%) | 67 (91·8%) |
| Mild | 9 (81·8%) | 42 (67·7%) | 51 (69·9%) |
| Moderate | 0 | 12 (19·4%) | 12 (16·4%) |
| Severe | 1 (9·1%) | 3 (4·8%) | 4 (5·5%) |
| Fiber necrosis | 6 (54·5%) | 44 (71·0%) | 50 (68·5%) |
| Coronary atherosclerosis | 7 (63·6%) | 31 (42·5%) | 38 (52·1%) |
| Interstitial hemorrhage | 7 (63·6%) | 27 (43·5%) | 34 (46·6%) |
| Epicarditis | 2 (18·2%) | 25 (34·2%) | 27 (37·0%) |
| Myocarditis | 3 (27·3%) | 11 (17·7%) | 14 (19·2%) |
| Secondary | 1 (9·1%) | 4 (6·5%) | 5 (6·8%) |
| Bacterium | 1 (9·1%) | 2 (3·2%) | 3 (4·1%) |
| Fungus | 0 | 1 (1·6%) | 1 (1·4%) |
| Chagas | 0 | 1 (1·6%) | 1 (1·4%) |
| Viral | 2 (18·2%)· | 7 (11·3%) | 9 (13·2%) |
| Focal | 1 (9·1%) | 3 (4·8%) | 4 (5·5%) |
| Multifocal | 1 (9·1%) | 3 (4·8%) | 4 (5·5%) |
| Abscess | 0 | 1 (1·6%) | 1 (1·4%) |
| **Immunohistochemistry (mean count per HPF)** | n=5 | n=19 | n=24 |
| CD68+ cells | 18·3 (13·2-20·0) | 14·3 (10·5-18·8) | 14·3 (10·6-19·5) |
| CD45+ cells | 3·4 (2·6-6·5) | 2·8 (2·0-4·3) | 2·8 (2·3-5·4) |
| CD57+ cells | 0·4 (0·2-0·6) | 0·3 (0·1-0·4) | 0·3 (0·1-0·5) |
| CD4+ cells | 0·8 (0·6-1·1) | 0·9 (0·5-1·5) | 0·8 (0·5-1·5) |
| CD8+ cells | 2·4 (1·2-5·2) | 0·8 (0·4-2·0) | 1·0 (0·4-2·3) |
| Positive YF viral antigen | 5 (100%) | 19 (100%) | 24 (100%) |
| Positive 1+ | 3 (60·0%) | 16 (84·2%) | 19 (79·2%) |
| Positive 2+ | 2 (40·0%) | 3 (15·8) | 5 (20·8%) |
| **RT-qPCR for YF** | n=11 | n=58 | n=69 |
| Positive | 11 (100%) | 55 (94·8%) | 66 (95·7%) |
| **Cardiac conduction system** | n=2 | n=6 | n=8 |
| Edema, hemorrhages and inflammatory infiltrate | 2 (100%) | 6 (100%) | 8 (100%) |
| SN artery fibrinoid necrosis | 0 | 2 (33·3%) | 2 (25·0%) |
| SN fibrosis | 1 (50·0%) | 1 (16·7%) | 2 (25·0%) |
| Conduction system fiber necrosis | 0 | 1 (16·7%) | 1 (12·5%) |
| Mycotic thrombus | 0 | 1 (16·7%) | 1 (12·5%) |
| Positive YF viral antigen | 1 (50·0%) | 3 (50·0%) | 4 (50·0%) |

^a^ Sex assigned at birth. CI: confidence interval; LV: left ventricle; HPF: high-power field; CD: cluster of differentiation; YF: yellow fever; RT-qPCR: reverse transcriptase quantitative polymerase chain reaction; YEL-AVD: yellow fever-associated viscerotropic disease; SN sinus node


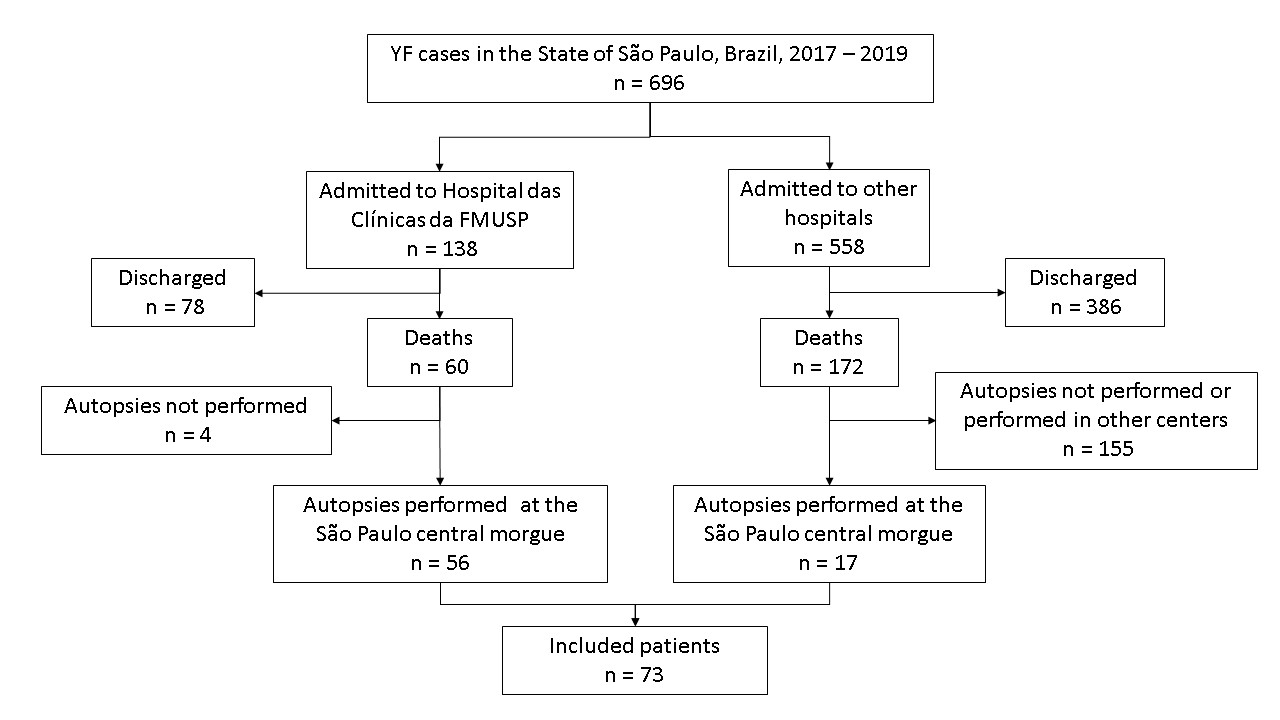


**Supplementary Figure 1 – Inclusion flow diagram.** YF: yellow fever; FMUSP: Faculdade de Medicina da Universidade de São Paulo.

**
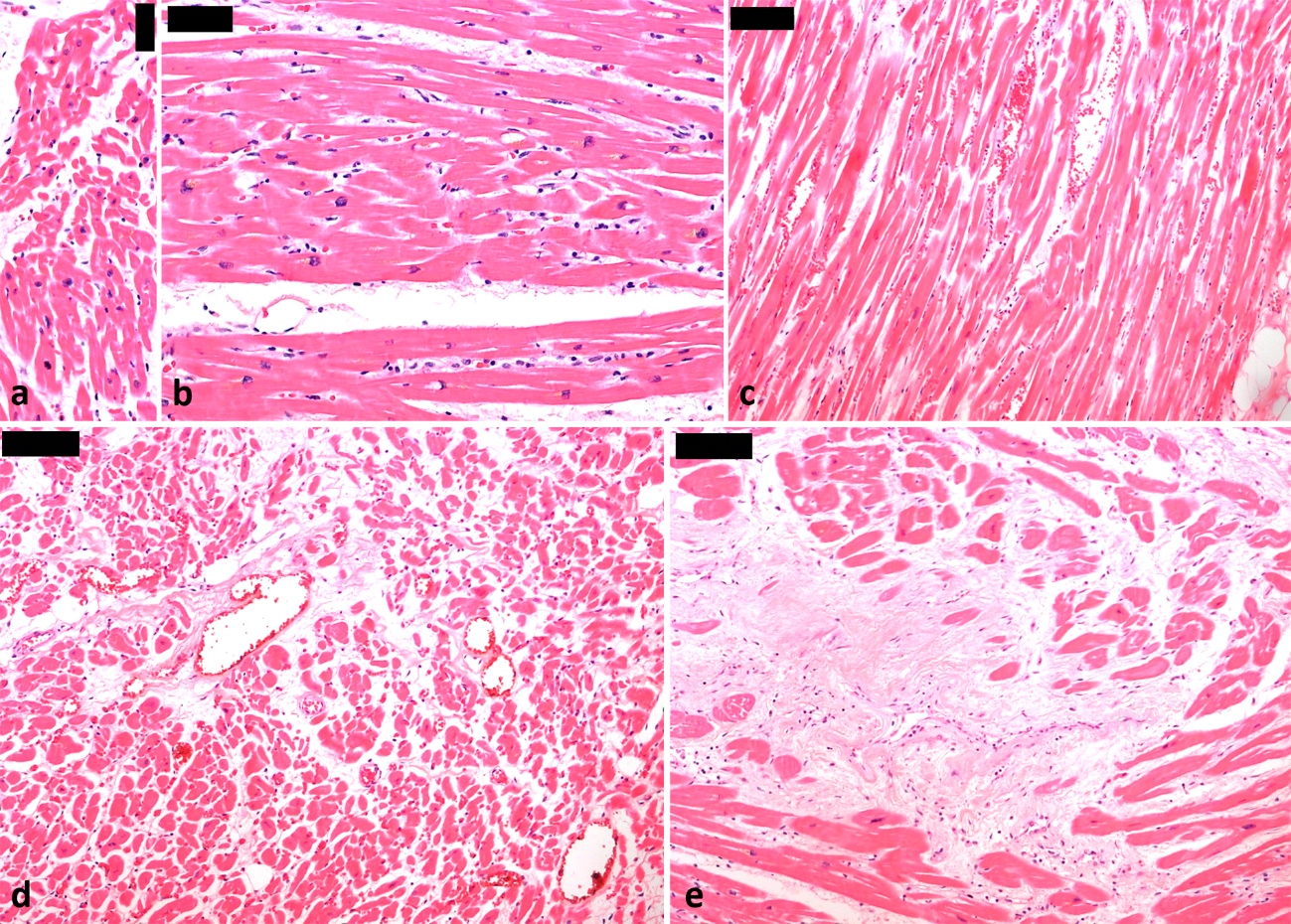
**

**Supplementary Figure 2 – Cardiac histopathology observed in control patients**. a. Hypertrophy of cardiomyocytes in a patient with hypertension. b. Hypertrophy of cardiomyocytes associated with mild interstitial mononuclear inflammatory infiltrate in a patient with hypertension and sepsis. c. Acute myocardial infarction, showing interstitial edema and hemorrhages, with ischemic fibers with thin, tortuous, and hypereosinophilic aspects and loss of nuclei. d. Recent area of acute myocardial infarction with neovascularization and loose colagenic interstitial matrix. e. Chronic ischemic myocardiopathy with focal area of dense interstitial scar, surrounded by hypertrophic cardiomyocytes. Magnifications: 200x (c,d,e); 400x (a,b). Scale bars: 50 µm (a,b); 100µm (c,d,e).


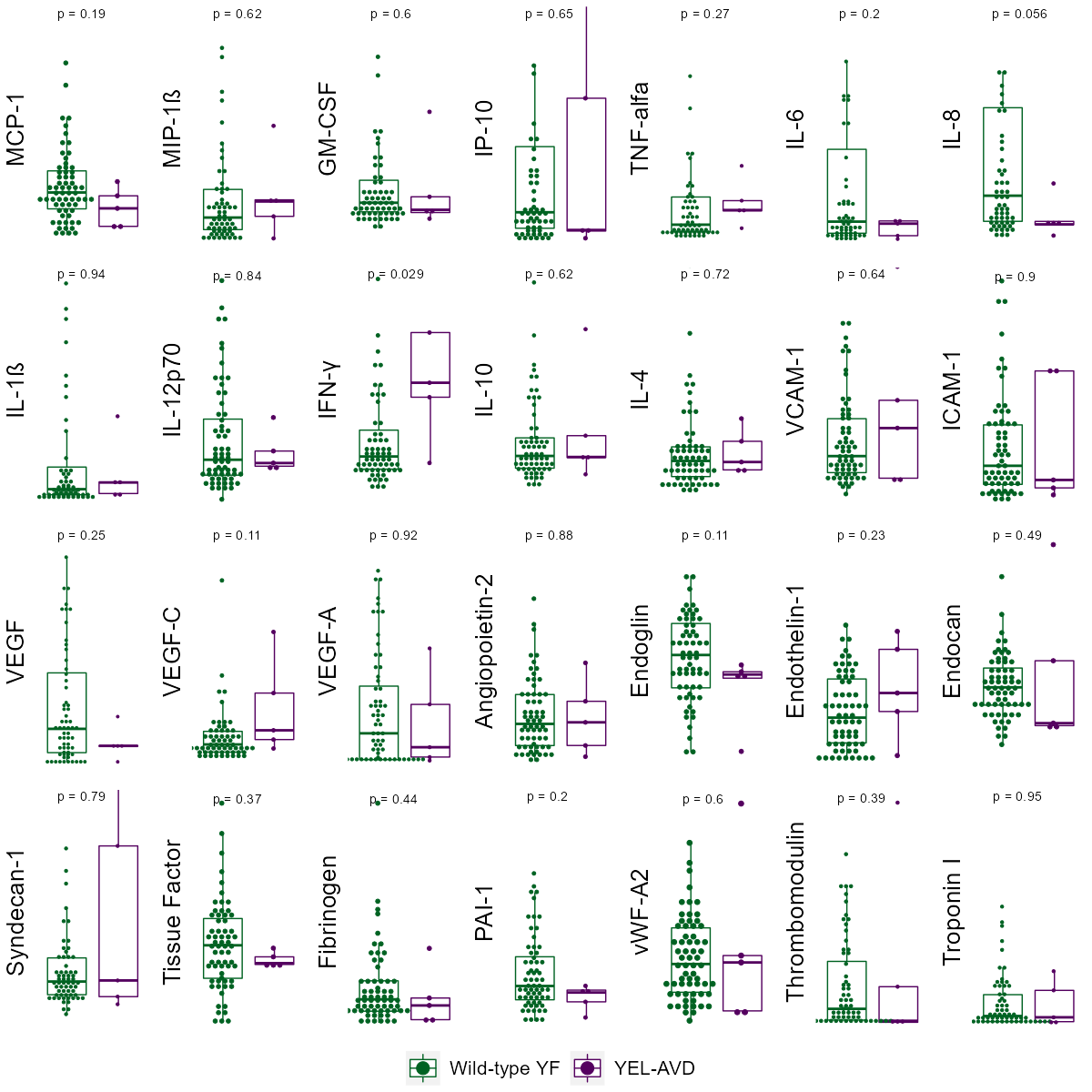


**Supplementary Figure 3 -** **Comparison of tissue proteomics analysis between yellow fever subgroups**. Cytokines and endothelial lesion biomarkers dosages performed through multiplex immunoassay on fragments of frozen myocardial tissue obtained from autopsy from 69 patients with yellow fever (YF), being 5 with yellow fever vaccine-associated viscerotropic disease (YEL-AVD) and 64 with wild-type yellow fever. All biomarkers dosages were normalized by total protein of each sample and only comparative data are shown. The Wilcoxon ranl-sum test was used for comparisons between groups. TNF: tumor necrosis factor; IFN-γ: Interferon-gama: interferon, IL: interleukin; MCP-1: monocyte chemoattractant protein 1; MIP1-β: macrophage inflammatory protein 1 - beta; IP-10: Interferon gamma-induced protein 10; GM-CSF: granulocyte and macrophage colony stimulating factor; VEGF: vascular endothelial growth factor; VCAM-1: vascular cell adhesion molecule 1; ICAM-1: Intercellular adhesion molecule 1; PAI-1: plasminogen activation inhibitor; vWF-A2: von Willebrand factor A2.
